# Unraveling the salt tolerance of Phi29 DNA polymerase using compartmentalized self-replication and microfluidics platform

**DOI:** 10.3389/fmicb.2023.1267196

**Published:** 2023-11-07

**Authors:** Yaping Sun, Danny Hsu Ko, Jie Gao, Kang Fu, Yaping Gao, Qiwen Zhang, Salem Baldi, Tao Hong, Igor Ivanov, Yun He, Hui Tian

**Affiliations:** ^1^Research Center of Molecular Diagnostics and Sequencing, Research Institute of Tsinghua University in Shenzhen, Shenzhen, China; ^2^Research Center of Molecular Diagnostics and Sequencing, Axbio Biotechnology (Shenzhen) Co., Ltd., Shenzhen, China

**Keywords:** compartmentalized self-replication (CSR), sequencing by synthesis (SBS), salt tolerance polymerase, droplet-based microfluidics, conservative amino acids

## Abstract

In Phi29-α–hemolysin (α-HL) nanopore sequencing systems, a strong electrochemical signal is dependent on a high concentration of salt. However, high salt concentrations adversely affect polymerase activity. Sequencing by synthesis (SBS) requires the use of phi29 polymerase without exonuclease activity to prevent the degradation of modified nucleotide tags; however, the lack of exonuclease activity also affects polymerase processivity. This study aimed to optimize phi29 polymerase for improved salt tolerance and processivity while maintaining its lack of exonuclease activity to meet the requirements of nanopore sequencing. Using salt tolerance compartmentalized self-replication (stCSR) and a microfluidic platform, we obtained 11 mutant sites with enhanced salt tolerance attributes. Sequencing and biochemical analyses revealed that the substitution of conserved amino acids such as G197D, Y369E, T372N, and I378R plays a critical role in maintaining the processivity of exonuclease-deficient phi29 polymerase under high salt conditions. Furthermore, Y369E and T372N have been identified as important determinants of DNA polymerase binding affinity. This study provides insights into optimizing polymerase processability under high-salt conditions for real-time polymerase nanopore sequencing, paving the way for improved performance and applications in nanopore sequencing technologies.

## 1. Introduction

Phi29 DNA polymerase, derived from *Bacillus subtilis phage* phi29, is widely used for DNA amplification via rolling-circle amplification (RCA) or multiple displacement amplification because of its high processivity, strand displacement activity, and high fidelity (Johne et al., 2009). Phi29 has been used for single-molecule sequencing in combination with nanopores via polymer-tagged nucleotides (Stranges et al., 2016). However, if polymer-tagged nucleotides are absent from a protection group, the strong exonuclease activity of phi29 polymerase leads to the degradation of the terminal oligonucleotides, ultimately resulting in no detectable sequencing signal. Hence, termination of exonuclease activity in phi29 is necessary when using polymer-tagged nucleotides. D12, E14, Y59, H61, N62, D66, F69, K143, Y148, Y165, and D169 have been reported to interact directly with single-stranded DNA (ssDNA), resulting in the formation of an active center for exonuclease activity (Esteban et al., 1994; Eisenbrandt et al., 2002; Wang et al., 2005; Perez-Arnaiz et al., 2009; Salas et al., 2016). D12A and D66A mutations have been shown to eliminate exonuclease activity (Del Prado et al., 2019). The double mutation dramatically reduces the acidity at the bottom of the active center, resulting in difficulties in repelling ssDNA to a stable position. Moreover, we found that the processivity of Phi29 with D12A was considerably lower than that of the wild type. Henceforth, it remains to understand how to enhance polymerase activity, while eliminating exonuclease activity.

In a single-molecule sequencing system, high salt concentration results in strong electrochemical signals. However, this environment is not suitable for polymerase activity, especially at 300 mM KCl or NaCl. The helix-hairpin-helix [(HhH)2] domain of *Methanopyrus kandleri* topoisomerase V was successfully fused to Taq and Pfu DNA polymerases to increase salt tolerance and processivity (Pavlov et al., 2002). Different HhH motifs may have different effects on extension rates. When the H and I motifs from the [(HhH)2] domain were coupled with phi29 polymerase, its DNA-binding affinity and salt tolerance were drastically enhanced (de Vega et al., 2010; Gao et al., 2021). However, the addition of a C-terminal domain results in a longer linker between the polymerase and the nanopore, thus hindering the capture of an electrochemical signal. Therefore, enhancing the salt tolerance of polymerases without external factors is a superior option for sequencing by synthesis (SBS). Altogether, attributes such as enhanced salt tolerance, processivity, and elimination of exonuclease activity are necessary for phi29 to excel in nanopore sequencing.

Compartmentalized self-replication (CSR) has been employed as a directed evolutionary strategy for thermostability, inhibitor tolerance, and reverse transcriptase activity of polymerases (Ghadessy et al., 2001; Povilaitis et al., 2016; Abil and Ellington, 2018). According to the findings of Ghadessy et al., Taq DNA polymerase obtained through CSR screening demonstrated an increased stability for longer durations at 95°C than wild type polymerases (Ghadessy et al., 2001). Through seven selection rounds of isothermal CSR (iCSR), Phi29 mutant variants’ thermostability were increased in the range of 40°C– 42°C (Povilaitis et al., 2016). Furthermore, CSR has been used to screen *Thermococcus kodakaraensis* (KOD) DNA polymerase with reverse transcriptase activity (Abil and Ellington, 2018).

In this study, the CSR for the selection of high salt concentrations is reported for the first time. The influence of a strong osmotic pressure environment on encapsulated droplets was studied using two methodologies. First, a modified CSR protocol is investigated, followed by a second study using a microfluidic platform. Based on the obtained results, a high-salt selection experiment was conducted for each method. A series of mutant sites were successfully identified that may improve salt tolerance and thereby enhance processivity. Evaluation of mutant sites through a combination of bioinformatics and biochemical analyses revealed that conserved amino acids with higher frequency play a significant role in salt tolerance and the processivity of polymerases. This is exemplified by G197, Y369, T372, and I378, which were mutated to G197D, Y369E, T372N, and I378R, respectively, for validation. The hybrid method presented in this paper not only ensures droplet consistency, but also empowers high-throughput screening capabilities for accelerating discoveries in various biological and biotechnological applications.

## 2. Methods

### 2.1. Libraries construction and stCSR

A comprehensive protocol detailing the library construction and stCSR is provided in the Supplementary file.

### 2.2. Identification of mutant sites

After the first screening, two enzymes (SgrAI and AsiSI) were used to digest the recovered RCA products, followed by purification of the target band (1.7 kb) via gel recovery. This target band was designated as a new template and amplified using library primers F and R (Supplementary Table S1). A second screening cycle was performed in which the amplified products from primers F and R were ligated with the same plasmid used in the first screening process. After two rounds of screening, the product recovery process was performed until the targeted band amplification by sequencing primers F and R was complete (Supplementary Table S1). The amplified product was ligated with a T-vector for further sequencing to identify mutant sites (Tsingke Bio, Guangzhou, China).

### 2.3. Plasmids preparation and protein purification

The coding sequences of the D12A, D12A_G197D, SZ_A, SZ_B, SZ_C, and D mutant variants were synthesized and cloned into pet30a using GeneScript (Nanjing, China). Wildtype sequence information was obtained from the NCBI database. The expression vectors pet30a-D12A, pet 30a-D12A_G197D, pet30a-SZ_A, pet30a-SZ_B, pet30a-SZ_C, and pet30a-SZ_D variants were transformed into chemically competent BL21 *Escherichia coli* cells (Tolobio, Wuxi, China) according to the manufacturer’s instructions.

The BL21 (DE3) strain of *E. coli* cells harboring the encoding polymerase plasmid were grown to OD600 = 0.6 in an LB medium at 37°C, and expression was induced by adding isopropyl-D-1-thiogalactopyranoside (IPTG) up to 0.6 mM concentration. The incubation temperature was decreased from 37°C to 16°C and protein production was carried out at 200 rpm for 20 h. After induction, the cells were harvested by centrifugation at 4000 × *g* and stored at −70°C. Semipurification was performed using a Ni Sepharose 6FF (17,531,801, Cytiva) gravity column (Borrego et al., 2022).

### 2.4. Rolling circle replication assay

Rolling circle amplification (RCA) was performed using two kinds of different templates: M13mp18 and CBT1. The M13mp18 template (N4040S; NEB, Ipswich, MA, United States) was prepared as described previously (Kong et al., 1993). M13mp18 as a 7,429 nt single strand circle DNA (sscDNA) was annealed to specific primers (Supplementary Table S1) at 95°C for 3 min and was left to cool at room temperature for 25 min. The CBT1 template was prepared as per a previously described protocol (Palla et al., 2021). Briefly, 51 nt CBT1 single-strand DNA (ssDNA) was hybridized with its primer (Supplementary Table S1). In order to retrieve sscDNA, CBT1 was circularized using T4 ligase at 16°C for three hours and digested by Exonuclease I and III to remove cyclic single strand DNA. The final primer-annealed circular CBT1 template was precipitated using 3 M sodium acetate and 100% ethanol, washed with 75% ethanol, and re-dissolved in nuclease-free water.

After template preparation, 10 nM of M13mp18 or CBT1 and 200 nM polymerases were mixed in a total of 20 μL solution at 30°C for 180 min and then stopped by adding 0.5 M EDTA. The RCA assay for each polymerase was performed in triplicates. These samples were then analyzed using 0.6% alkaline agarose gel electrophoresis (Gao et al., 2020).

The GeneRuler High Range DNA Ladder (10.1 kb–48.5 kb, SM1351; Thermo Fisher Scientific, United States) and 1 KB plus (0.3 kb-10 kb, P21119, TransGen Biotech Co., Ltd., Beijing, China) were used as DNA markers. A peristaltic pump (BT100-1 L; Longerpump, Baoding, China) was used to circulate the running buffer, preventing elevated temperatures during the gel electrophoresis process. After 2–3 h of electrophoresis, the gel was stained with SYBR Gold, and the concentrations and molecular weights of the RCA product were assessed using AzureSpot.

### 2.5. qRCA assay

Primer-annealed circular CBT1 (Supplementary Table S1) was used as a template in the qRCA assay because the prepared M13mp18 template was too long to obtain a differential fluorescence signal. Initially, 200 nM polymerase was mixed with 20 nM template and 2 μM SYTO^®^ Green-Fluorescent Nucleic Acid Stains (S34854; Thermo Scientific, United States) in 50 μL reaction buffer. The reaction buffer was 1*phi29 buffer (B0269S, NEB, Ipswich, MA, United States), 50 μM dNTPs, and 0.1 mg/mL BSA (B9000S, NEB, Ipswich, MA, United States). The samples were briefly centrifuged and placed in the thermal module of the AriaMx Real-time PCR System (G8830A, Agilent, United States) as soon as possible. The fluorochrome was selected as SYBR, and the temperature was set at 30°C for 3 h. Polymerase activity in different salt concentration conditions was tested. Each polymerase was used in triplicate.

### 2.6. Template binding affinity of DNA polymerase and Seq99A

Seq99A, a double-hairpin ssDNA (Supplementary Table S1; Supplementary Figure S1), was used as the template for this binding assay. Before use, Seq99A synthesized by Genescript (Nanjing, China) was heated in a metal bath at 95°C for 5 min and then returned to room temperature for 30 min. Subsequently, 10 nM prepared Seq99A template was mixed with 100 nM polymerase (1:10) in 10 μL incubation buffer (20 mM HEPES, 1 mM MgCl2, and 4 mM DTT buffer). The mixture was incubated at 30°C for 10 min. Next, 150 and 225 mM KCl were added to the above system for 10 min. After which, 8 μL sample and 2.5 μL high density TBE loading were run in 4–20% TBE gel (Thermo Fisher, United States) for 50 min with 120 V condition. The gels were stained with SYBR GREEN for 30 min. The binding ability was assessed according to band migration in the EPI Blue model using AzureSpot.

### 2.7. Droplet-based microfluidic experiments

Single *E. coli* cells with expressed proteins were encapsulated into water in oil droplets using a commercial microfluidic chip (Zhong Xin Qi Heng, China) catalogue number ZX-LD-40 equipped with a droplet formation entrance of 50 μm. In the continuous oil phase, 10 mL of premixed oil with 200 μL ABIL EM 90 (Degussa), 5.5 μL Triton X-100 (T0694, Amresco, Solon, United States), and 9,800 μL Mineral oil (M5904-500ML, Sigma-Aldrich, St. Louis, MO, United States) was used, whereas a 3 mL RCA reagent comprised of 1* Tango buffer, 1 mM Exo-resistant random primer, 10 μM of plasmid-specific primers, 10 mM dNTP analogs, 50 μL lysed *E. coli*, and 300 mM KCl was used in the water phase. In the first set of control experiments, single-cell bacteria were excluded from the water phase to determine and optimize the flow rate conditions and their respective droplet sizes. Next, new fluidic tubing of the same length was installed before the single-cell encapsulation experiment.

Freshly prepared oil and reagents were loaded into two separate 10 mL syringes and driven by independent syringe pumps (Lead Fluid, TYD02-02, China). The water phase flow rate was kept constant at 8 μL/min, while varying different oil flow rate conditions employed the means to manipulate droplet sizes. Two experimental conditions with three replicates were used. The oil phase flow rate was kept at 32 μL/min and 22 μL/min to generate 30 μm and 50 μm droplets, respectively.

Droplets were observed under a SOPTOP CX40M microscope (Yuyao, Zhejiang, PR, China). Bright-field images were captured using a high-speed camera at 100 FPS (Laite, LF50, China) at 20 × magnification. Droplet size and estimated collection were analyzed using ImageJ software.

### 2.8. Bioinformatics and structural analysis

One hundred homologous genes of phi29 DNA polymerase were obtained using NCBI BLAST. Conservative amino acids were calculated and plotted using the R package ggmsa (Zhou et al., 2022). HotSpot Wizard 3.0 was used to design mutant sites, which could provide information about the frequency of amino acid occurrences and identify deleterious and beneficial mutations (Sumbalova et al., 2018). The websites could score the mutation, which could help us choose the kinds of amino acids present.

The Phi29 polymerase structure was downloaded from the Protein Data Bank (Berman et al., 2000, 2007). The structures of the mutant variants were predicted using Alpha Fold2 (Cramer, 2021). The structure and electrostatic potential maps of the surface were created and analyzed using PyMOL (Hagemans et al., 2015; Kagami et al., 2020).

## 3. Results

### 3.1. Optimizing the CSR process for salt tolerance selection

**Figure 1:**
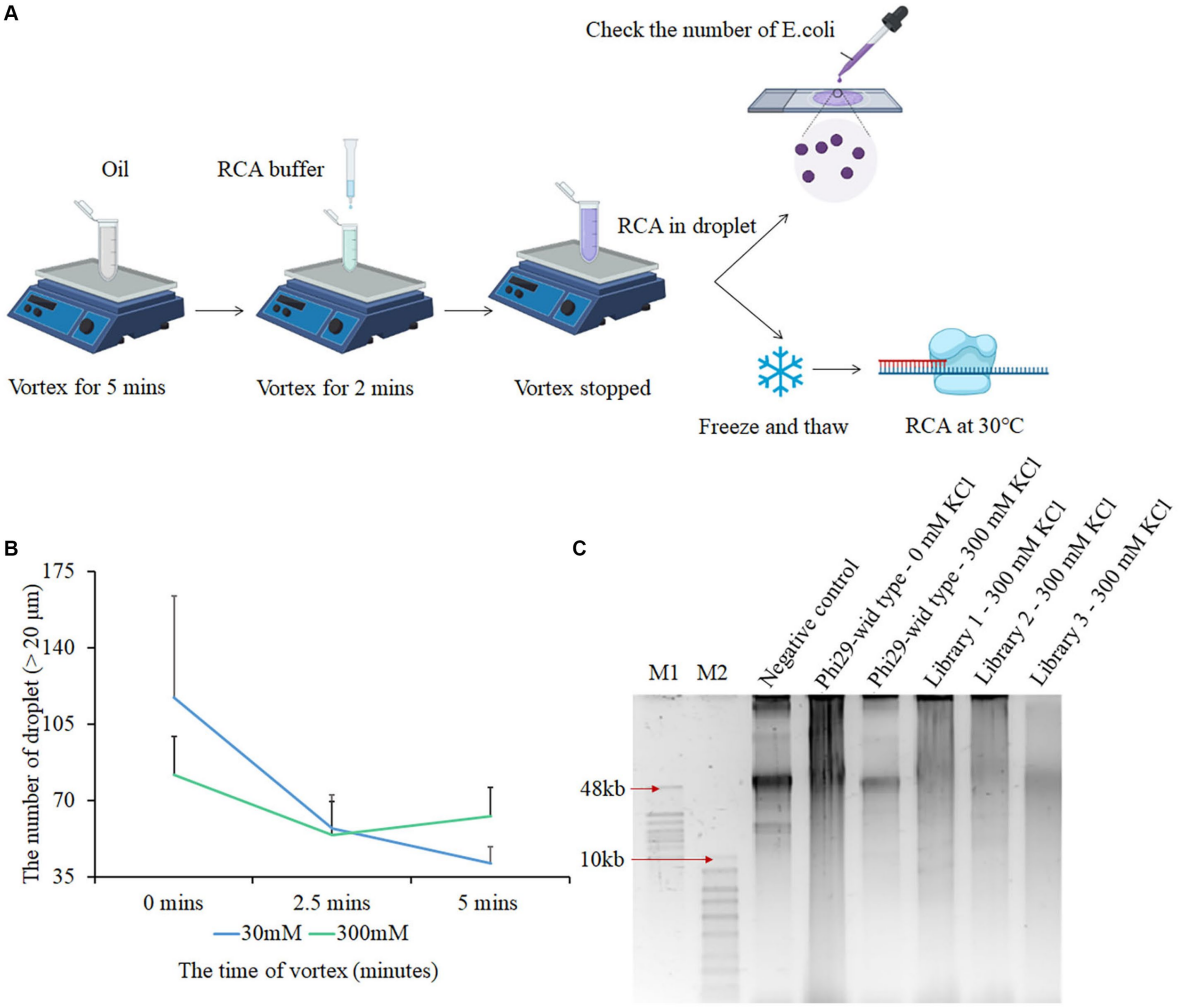
Screening polymerases with salt tolerance using compartmentalized self-replication (CSR) method. **(A)** Process of salt tolerance compartmentalized self-replication (stCSR). **(B)** Relationship between the final vortex time and the size of droplets. After mixing RCA reagents with oil in 0 mM or 300 mM KCl condition, the third vortex was performed at different times. Droplet sizes larger than 20 μm were observed using a 10× magnification optical microscope. **(C)** Results based on the first round of RCA products screening. Lane 1 refers to a negative control using *E. coli* with plasmid backbone in salt-free condition; Lanes 2 and 3 as a positive and negative control, respectively, refer to *E. coli* with phi29 expression plasmid in salt-free and 300 mM KCl condition, respectively. Lanes 4, 5, and 6 refer to *E. coli* with mutant library 1, 2, and 3 expression plasmids in 300 mM KCl, respectively. M1 refers to GeneRuler high range DNA ladder, and M2 refers to 1 KB plus ladder.

To directly screen for polymerase mutants with strong salt tolerance traits, we optimized phi29 polymerase mutant libraries, droplet formation conditions, and the CSR product recycling process, defined as salt-tolerant compartmentalized self-replication (stCSR). We used two methods to obtain three libraries with distinct mutation sites. The first approach used error-prone PCR kits from Beyotime Biotechnology (Song et al., 2021), which regulate the number of mutant sites by varying the number of templates. The second method involves adjusting the content of MnCl_2_ to accommodate the number of mutant sites (Fujii et al., 2006). To calculate the number of mutant sites and mutant variants in each library, three parameters were considered: (1) Efficiency of transformation and ligation (50000–100,000 monoclones/100 ng ligation system), (2) efficiency of positive clones (95%–100%), and (3) number of mutant variants by sequencing the positive clones. By considering each of the subsequent results, mutant sites ranging from 6 to 14 and 10^6^ mutant variants in each library were determined. The number of mutants and mutant sites in the three libraries is presented in Table 1. Despite the presence of various mutant sites, they did not appear to interfere with protein expression (Supplementary Figure S2A). Water-in-oil emulsions were prepared according to a previously described protocol. This involved vortexing the oil for 4–5 min, followed by the dropwise addition of PCR reagents. The final mixture was vortexed for an additional 5 min to complete the preparation step (Ghadessy et al., 2001). In order to encapsulate a single bacteria cell in the emulsion, the size of the droplet is limited to 20–30 μm. However, in a high-salt environment, the high osmotic pressure results in the formation of smaller droplets. Therefore, it is difficult to package both *E. coli* cells and PCR reagents as well as to form a suitable emulsion using the protocol described above. A modified version of the protocol was developed, in which the last vortex step was removed. This modification resulted in the retrieval of suitable droplet sizes (Figure 1A). The droplet size decreased as the reagent mixture was vortexed for longer durations. Furthermore, prolonged vortexing resulted in a higher incorporation of impurities within the droplets (Figure 1B; Supplementary Figure S2B). Subsequently, RCA takes place within the oil droplet at 30°C for 3 h (Figure 1C). Based on the polyacrylamide gel electrophoresis results, the presence or absence of DNA bands served as a reliable determinant for a negative control test. The presence of a DNA band, specifically at the 50 kb position, is directly associated with the genomic characteristics of *E. coli*, which indicates that CSR amplification was unsuccessful. Distinct amplification product signals were detected in each of the three conditions examined, including a negative control without polymerase, a positive control using salt-free phi29, and a high-salt environment library. As shown in Figure 1C, this suggests that the regulatory sites for salt tolerance exist within these libraries. Notably, the length of the amplification product was affected when isothermal amplification was performed in a CSR system for an extended period exceeding 3 h, leading to a remarkable reduction in the length of the final product. This is likely due to the reduction in polymerase activity in the system, which increases exonuclease activity.

**Table 1:** The number of mutant sites and mutant variants in the three libraries.

Libraries	Number of mutant sites/1.7 kb	Number of mutant variants
1	6–10	10^6^
2	8–10	10^6^
3	12–14	10^6^

**Figure 2:**
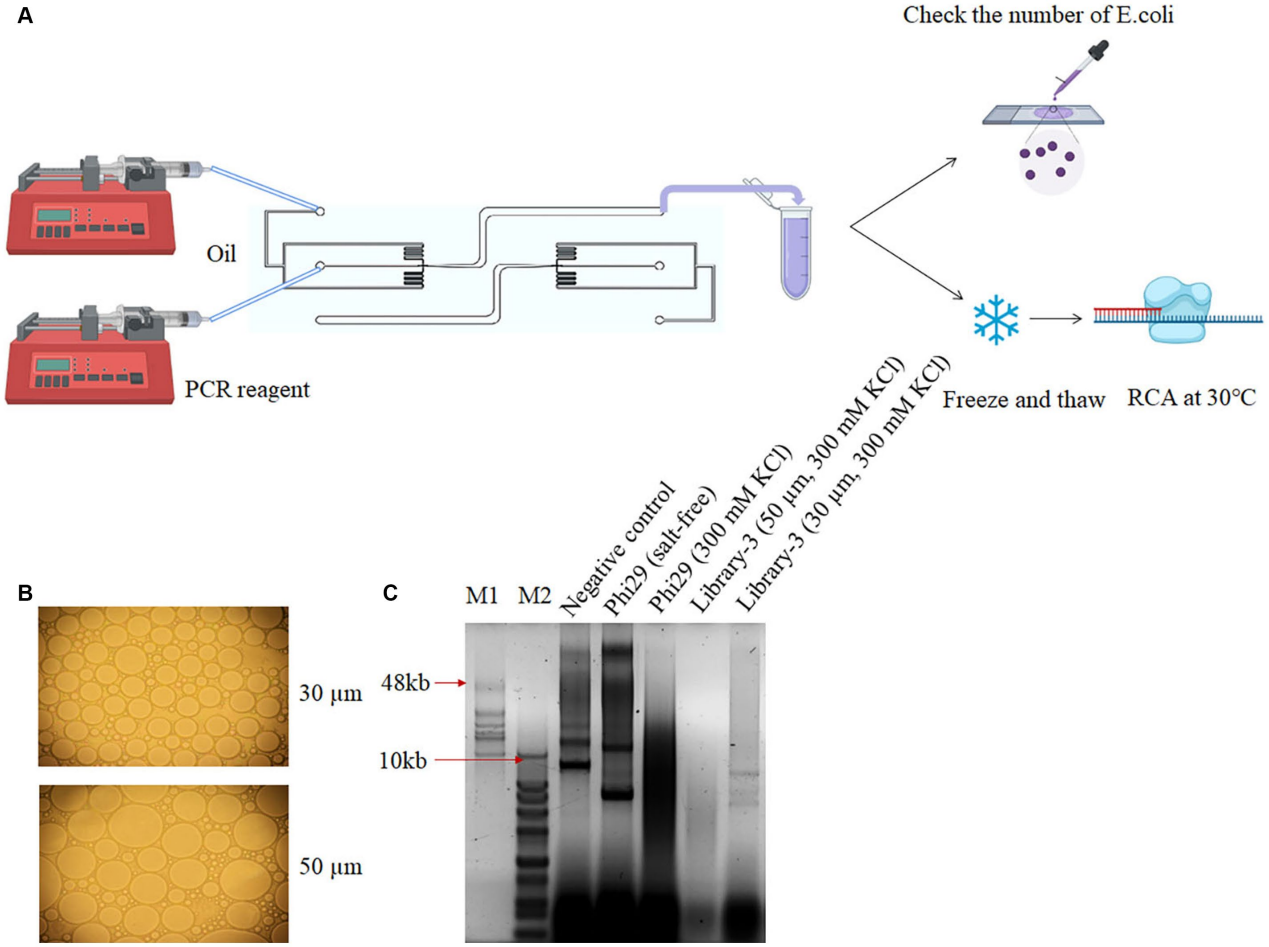
Screening polymerases with salt tolerance using droplet-based microfluidics. **(A)** Simple schematic of droplets generated using a droplet-based microfluidics system. **(B)** Microscopic observation of the collected droplets under various flow rates of RCA reagent and oil conditions. Two optimized flow parameters generated 30 μm and 50 μm droplets. **(C)** Screening of RCA products post microfluidics collection experiment. Lane 1 refers to a negative control using *E. coli* with plasmid backbone in salt-free condition. Lanes 2 and 3 as positive and negative controls, respectively, refer to *E. coli* with phi29 expression plasmid in salt-free and 300 mM KCl condition, respectively. Lanes 4 and 5 refer to *E. coli* with mutant library 3 expression plasmids and 300 mM KCl in 30 μm and 50 μm droplets, respectively. M1 refers to GeneRuler high range DNA ladder, and M2 refers to 1 KB plus ladder.

The overall droplet size was found to be nonuniform when the vortexing technique was employed. An alternative approach utilizing a droplet-based microfluid platform was then investigated to increase the droplet uniformity. The water phase containing both *E. coli* cells and the desired PCR reagent was mixed homogenously. The flow-focusing microfluidic chip comprising two inlet microchannels was used to introduce water-phase reagents at inlet (a) and oil at inlet (b) using independently controlled syringe pumps, as depicted in Figure 2A. Flow focusing between the two-phase occurred at an outlet of 50 μm diameter, before extending to a collector channel. Droplet size was manipulated by varying the flow rate of both phases, and the encapsulation of bacterial cells was evaluated. Experimental conditions were optimized to generate droplets of two different sizes: 30 μm and 50 μm in diameter. The number of active cells in the droplets was determined using an optical microscope at 50× magnification. Through microscopic examination, we found more than 10 *E. coli* cells trapped inside a 50 μm diameter droplet, while less than 3 *E. coli* cells were found inside a 30 μm diameter droplet (Figure 2B). A total of 10^6^ droplets were enriched and subjected to amplification at 30°C for 3 h. Based on the results of polyacrylamide gel electrophoresis, the number of products generated from 30 μm droplets was higher compared to that of 50 μm (Figure 2C). We thus concluded that 30 μm was a suitable size for stCSR. This excellent approach solves the disadvantages of using a multistep vortexing protocol; thus, a continuous-flow droplet-based microfluidic system was integrated into the development of homogenous droplets for high-throughput screening of DNA polymerases.

### 3.2. The sequence conservation and amino acid frequency analysis of mutant sites

**Figure 3:**
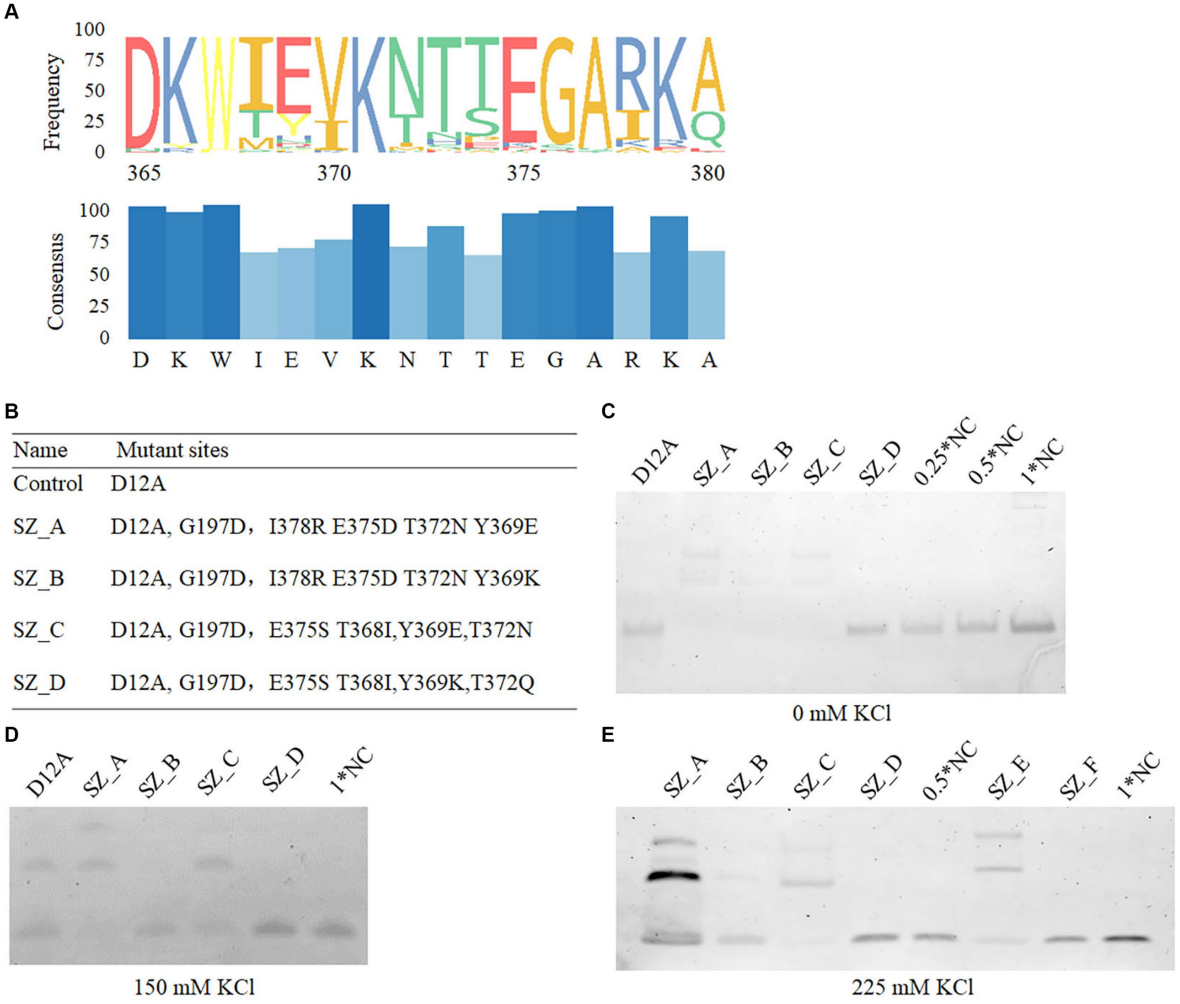
The binding affinity of mutant variants. **(A)** The conservative amino acids statistics in the 365–380th position of phi29. The conservative amino acids were calculated using 100 homologue genes of phi29 from NCBI. **(B)** All mutant sites in mutant variants. **(C–E)** The binding affinity of D12A, SZ_A, SZ_B, SZ_C, and SZ_D in salt free, 150 mM, and 225 mM KCl conditions, respectively. NC refers to a negative control. A total of 10 nM prepared Seq99A template was incubated with 100 nM polymerase in 10 μL binding buffer (20 mM HEPES, 1 mM MgCl_2_, and 4 mM DTT buffer) at 30°C for 10 min. 0 mM **(C)**, 150 mM (D), and 225 mM KCl **(E)** were mixed, respectively. 0.5*NC and 1*NC refer to 0.05 μM and 0.1 μM seq99 templates without polymerases, respectively.

After two cycles of screening, 116 monoclonal bacterial cells were sequenced. Most sequences were terminated upon encountering the stop codon at the halfway point, leading to the final identification of 11 monoclones comprising a total of 85 mutant sites. Notably, 10 mutant sites, namely Y109, G197, K361, T368, Y369, T372, E375, I378, L412, R496, and K538 were repeated twice. To further analyze and explore the suitability of amino acids among the identified mutant sites, HotSpot Wizard, a computational tool, was used to examine the stability of these amino acids. This analysis provided valuable information, including the frequency of amino acids identified and their respective potential for catalytic activity after mutation. For a conservative analysis, 100 homologue gene sequences of phi29 from the NCBI BLAST function were analyzed. Table 2 summarizes the conserved amino acids and their corresponding scores based on the Hotspot Wizard and the 100 homologous gene sequences. According to the above homologous gene analysis, many classical amino acids facilitate polymerase thermostability (Povilaitis et al., 2016). For example, aspartic acid is the most conserved amino acid in the 197th position of the 100 homologous genes for phi29 and scored 87 in the HotSpot Wizard. Glutamic acid, glycine, and aspartic acid were the first, second, and third most frequent amino acids at position 197, respectively. Mutation of G197D has demonstrated the ability to enhance the temperature tolerance of phi29 from the typical stable temperature of 30°C to 40–50°C. However, whether this enhances salt tolerance remains unclear. We hypothesized that conserved amino acids with higher frequencies may contribute to the thermostability, salt tolerance, and processivity of polymerases. Y369, T372, E375, and I378 are located at the edge of the substrate entrance; however, whether their mutations would be advantageous for salt tolerance needs to be investigated. With respect to stability and conservative analysis, Y369E, T372N, E375E, and I378R all exhibited better conservative traits (Figure 3A), whereas Y369K, T372E, E375Q, and I378L exhibited the highest frequency, and Y369K (88), T372Q (100), E375N (100), and I378R (100) scored the highest in terms of stability according to the HotSpot Wizard. Asn, the amino acid with the highest frequency at position 375, has been reported to possess elevated salt tolerance (Gao et al., 2021). Although both Gln and Asn exhibited a similar charge characteristic, it is worth noting that both Asp and Asn have shorter side chains than Glu and Gln. Whether the shorter Asp and Asn side chains at position 375 enhance the salt tolerance of polymerases remains unknown. Based on the above statistical analyses, four mutant combinations, SZ_A, SZ_B, SZ_C, and SZ_D, were designed and evaluated.

**Table 2:** The sequence conservation and amino acid frequency analysis of candidate key sites.

	Frequency (first)/Score	Frequency (second)/Score	Frequency (third)/Score	Frequency (fourth)/Score	Conservative amino acid and frequency from 100 homologue genes
Y109	G/81	Y	T/86	R/84	V(61/100)
G197	E/85	G	D/87	T/84	D(65/100)
K361	K	D/88	R/84	N/78	K(92/100)
T368	M/88	I/84	K/86	T	I(63/100)
Y369	K /88	E/84	Y	R/86	E(66/100)
T372	E/85	K/100	T	N/84	N(67/100)
E375	Q/100	T/84	S/86	D/87	E(88/100)
I378	L/87	K/100	I	E/84	R(63/100)
L412	L	V/87	I/84	T/78	V(64/100)
R496	R	K/81	G/50	×	R(97/100)
K538	K	A/81	Y/84	R/86	K(94/101)

“×” refers to the absence of available frequency statistical analysis.

In polymerase-nanopore sequencing systems, artificial substrates modified by oligonucleotides have been extensively used as substitutes for natural bases. The strong exonuclease activity of Phi29 polymerase degrades oligonucleotides. To eliminate the exonuclease activity, we incorporated D12A into the four mutant combinations. A separate mutant variant containing only D12A was used as a control.

In this study, five mutant variants were constructed, where the D12A mutant site was included to decrease exonuclease activity (Figure 3B). SZ_A and SZ_C contained the most conserved amino acids, T368, Y369, T372, and I378. The frequency of Lys was the highest among amino acids at position 369. To validate its function, we mutated Y369K to SZ_B. Ser at position 375 of phi29 has been reported to possess elevated salt tolerance (Gao et al., 2021), and Asn and Gln have similar charge characteristics at position 372. Hence, the addition of E375S to SZ_C and E375S and T372Q to SZ_D were studied to evaluate their respective functions.

### 3.3. Amino acid mutation at Phi29 DNA polymerase substrate entrance for the enhancement of template and primer binding affinity

To investigate the impact of high salt concentration on the binding affinity of template primers and polymerases, the five polymerases, D12A, SZ_A, SZ_B, SZ_C, and SZ_D, were examined using the Seq99 template at varying salt concentrations. Under the same salt-free environment, as shown in Figure 3C, SZ_A, B, and C exhibited a higher affinity for binding ssDNA sequences than SZ_D. Under a 150 mM electrolyte environment, the binding affinity of SZ_B was notably lower (Figure 3D). As the salt concentration increased to 225 mM, the binding affinity of SZ_A was further reduced for the ssDNA template and primer (Figure 3E). However, SZ_C showed a constant binding affinity for both the ssDNA template and primer. These results suggest that both SZ_A and SZ_C have higher binding affinities than the other mutant polymerases.

### 3.4. Amino acid mutation at Phi29 polymerase substrate entrance for salt tolerance enhancement

**Figure 4:**
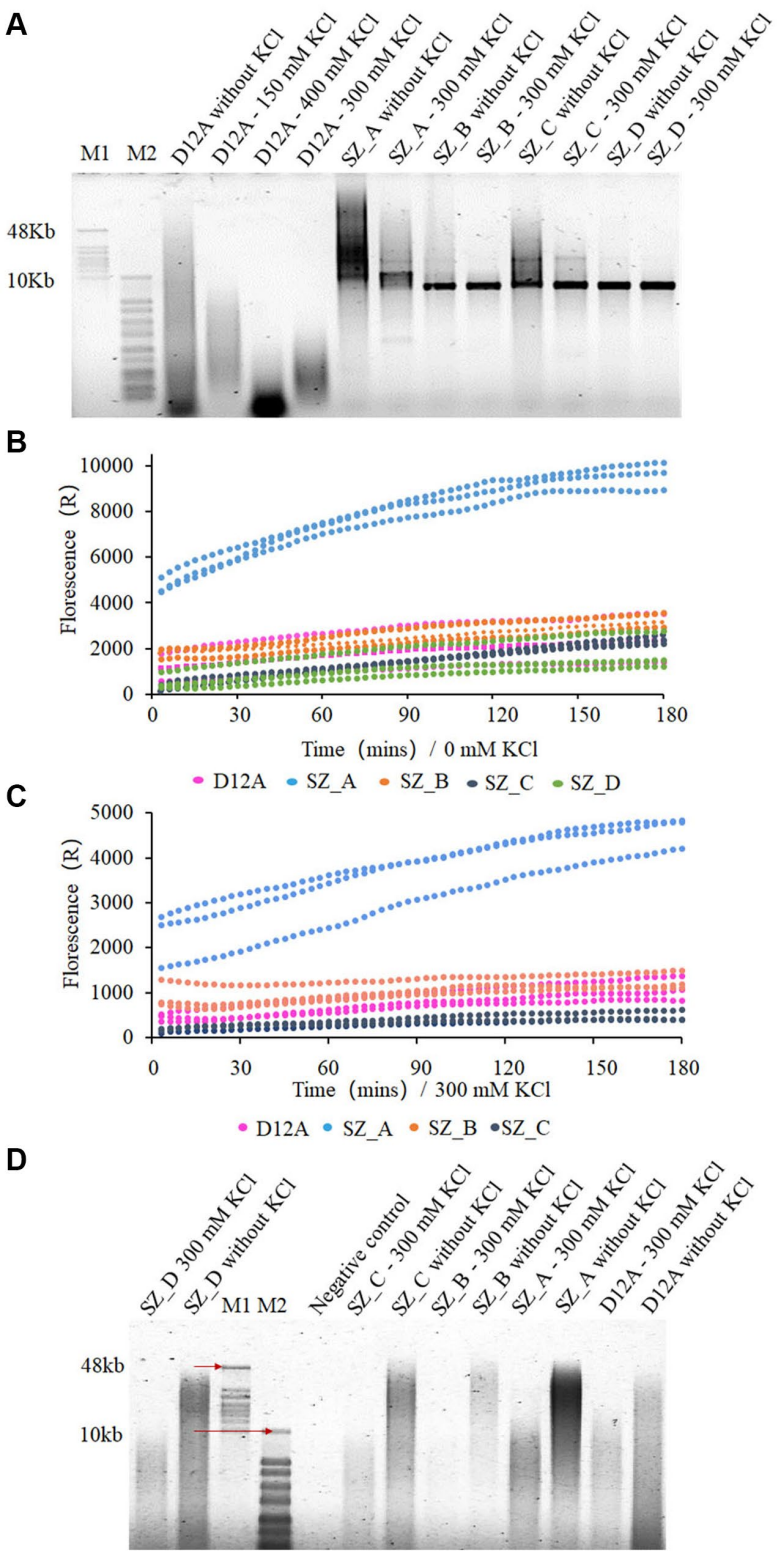
SZ_A with stronger salt tolerance and processivity compared with SZ_B, SZ_C, and SZ_D. **(A,D)** The extension experiments of mutant variants using prepared M13mp18 template **(A)** and CBT1 template **(D)**. Two hundred nM polymerases and 10 nM template were mixed with different salt concentration at 30°C for 180 min. **(B)** and **(C)** qRCA assays of mutant variants in 0 mM and 300 mM KCl, respectively. Two hundred nM polymerase was mixed with 20 nM CBT1 template and 2 μM SYTO in 50 μL reaction buffer at 30°C for 180 min. M1 refers to GeneRuler high range DNA ladder, and M2 refers to 1 KB plus ladder. Negative control refers to templates without polymerases in the reaction buffer.

To test the salt tolerance of the mutant variants, processivity assays were performed at different salt concentrations and template conditions. When the circular single-stranded DNA M13mp18 (7,249 bp) was used as a template, SZ_A and SZ_C exhibited increasingly longer products than D12A without salt. Moreover, the size of the product was centralized in the range of 10–60 kb, whereas D12A appeared as a dispersive band. Both SZ_B and SZ_D showed weaker polymerase processivity under salt-free conditions (Figure 4A). In comparison, both SZ_A and SZ_C results indicated extensive salt tolerance when compared to D12A, SZ_B, and SZ_D. Particularly for SZ_A, the dispersive band of the product was in the range of 7–50 kb under 300 mM KCl but retained the ability to accommodate longer DNA fragments under high salt conditions. To further assess polymerase activity, real-time quantitative RCA (qRCA) was used for the discussed variants. The fluorescence intensity was greater for SZ_A than for the other polymerases using CBT1 as a short single-stranded template under salt-free and high-salt concentration conditions (Figures 4B,C). Under salt-free and high-salt concentration conditions, despite similar fluorescence intensities observed at 180 min for SZ_C and D12A (Figures 4B,C), SZ_C demonstrated a better processivity response than D12A, as shown in Figure 4D. The qRCA is an indicator of the total amount of product generated, making it difficult to assess the processivity of the discussed polymerases. In conclusion, SZ_A exhibited better salt tolerance and processivity than the other treatments.

To further validate the function of these sites located at the edge of the substrate entrance, another mutant variant with D12A and G197D, was designated control 2. We compared the processivity of SZ_A with that of control 2 under salt-free and high-salt concentration conditions. SZ_A showed stronger salt tolerance and better processivity than control 2 (Supplementary Figure S3). This indicates thatY369E, T372N, E375D, and I378R are critical mutation sites for improving salt tolerance. Moreover, the D12A_G197D mutant variant produced a longer product than D12A alone (Figure 4A; Supplementary Figure S3). G197D not only improved the thermostability but also enhanced salt tolerance.

**Figure 5:**
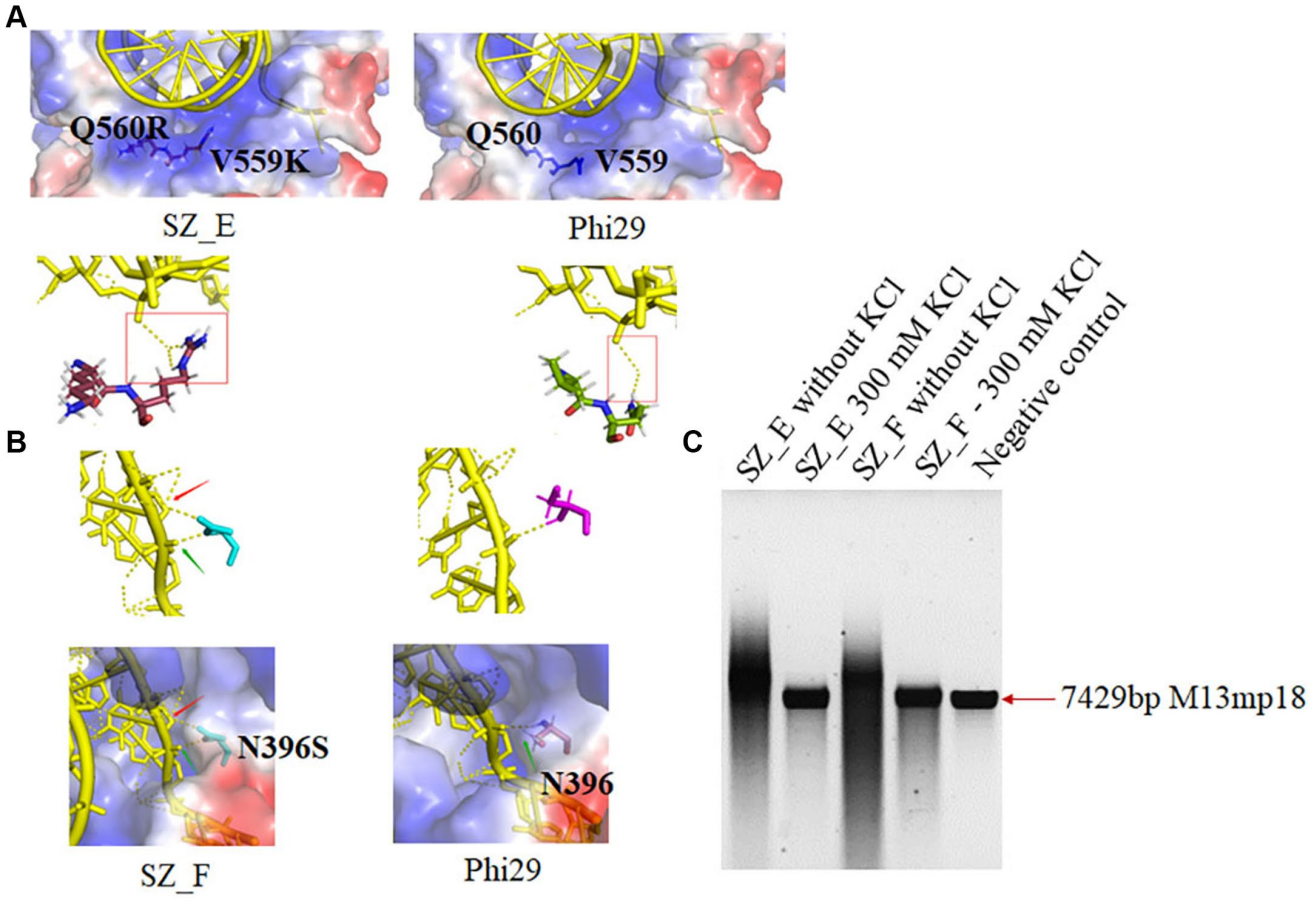
The structure and salt tolerance analysis of SZ_E and SZ_F. **(A)** The chemical structure in positions 559 and 560 of Phi29 (V559 and Q560) and SZ_E (K559 and R560) are illustrated. Yellow color: templates; brown color: K559 and R560; and green color: V559 and Q560. **(B)** The chemical structure in position 396 of Phi29 (N396) and SZ_F (S396) are also illustrated. Cyan color: S396 and purple color: N396. **(C)** Extension experiments of SZ_E and SZ_F. Two hundred nM polymerases and 10 nM template were mixed with different salt concentration at 30°C for 180 min. Negative control refers to M13mp18 (7,429 nt) template without polymerase.

Based on binding affinity analysis and amplification assays, we hypothesized that binding affinity does not correlate proportionally with improved polymerase activity, including extension and processivity, under high-salt conditions. For example, the binding affinity of SZ_C was higher than that of SZ_ A. However, SZ_A exhibited better salt tolerance and processivity than SZ_C. The effect of binding affinity on processivity under high-salt conditions was explored using two additional mutant variants. Conservative analysis suggested that Lys at position V559 and Arg at position Q560 were the most conserved amino acids among the 100 homologous genes. We combined D12A, G197D, V559K, and Q560R into SZ_E and predicted that it would possess a stronger binding affinity. As shown in Figures 3E, 5A, V559K and Q560R, in close proximity to the template, showed stronger binding interactions with the template. SZ_E shares a binding affinity similar to that of SZ_C. N396 was also considered because of its indirect and direct interactions with the ssDNA template in an aqueous solution. N396S was incorporated into SZ_F to reduce its indirect interactions with ssDNA (Figure 5B). As shown in Figure 3E, SZ_F showed a weaker binding affinity. The binding results of SZ_E and SZ_F were consistent with our predictions (Figure 3E). Through processivity analysis, SZ_E did not display significant salt tolerance or better processivity owing to its stronger binding affinity, and SZ_F with weak binding affinity showed reduced salt tolerance and processivity. In conclusion, a weaker binding affinity was not helpful for salt tolerance or the processivity of polymerases. A significantly stronger binding affinity also hinders the movement of the template and primer, resulting in weak processivity of the polymerases (Figure 5C).

## 4. Discussion

CSR has been extensively employed in DNA polymerase evolution to enhance thermostability, mitigate the effects of inhibitors, and obtain desirable traits beyond DNA polymerase activity. The compartmentalized self-tagging (CST) method, a derivative of CSR, was designed to select polymerases capable of incorporating modified nucleic acids (Pinheiro et al., 2012). High-temperature isothermal compartmentalized self-replication (HTI-CSR) is another derivative of CSR that has been used to screen for a more thermostable strand-displacing polymerase (Milligan et al., 2018). To facilitate a more efficient approach for screening polymerases with improved salt tolerance, a novel procedure called salt tolerance compartmentalized self-replication (stCSR) was developed based on the CSR technique. Because of the osmotic pressure from high salt concentrations, the size of the oil droplets and the activity of *E. coli* cells were reduced, negatively affecting the recycling of DNA products. Therefore, optimizing the size and uniformity of oil droplets *in vitro* is far more efficient than using conventional approaches. The droplet-based microfluidic technique not only enables the encapsulation of RCA reagents and *E. coli* cells, but also promotes the number of recycled products as a means to identify mutant sites via a high-throughput technique.

**Figure 6:**
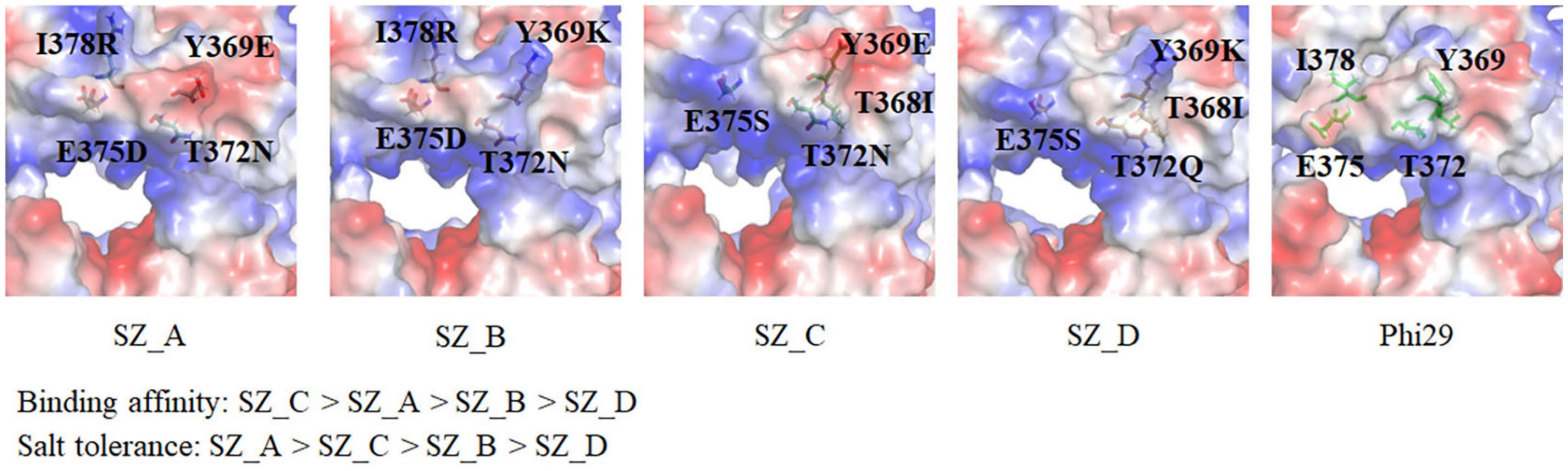
Surface charge analysis and chemical structures of mutant amino acids in SZ_A, SZ_B, SZ_C, and SZ_D. Based on the biochemical analysis, the orders of binding affinity and salt tolerance are as follows: SZ_C > SZ_A > SZ_B > SZ_D and SZ_A > SZ_C > SZ_B > SZ_D. A comparison of mutant sites SZ_A (Y369E, T372N, E375D, and I378R) and SZ_B (Y369K, T372N, E375D, and I378R) reveals that the conservative amino acid Y369E in SZ_A exhibits better salt tolerance performance due to an extra negative charge at the edge of the substrate entrance. This trait is also observed in SZ_C (T368I, Y369E, T372N, and E375S) and SZ_D (T368I, Y369K, T372Q, and E375S).

Eleven mutant sites for salt tolerance were identified through screening. Parameters such as potential catalytic activity, frequency, and conserved amino acids were considered for the five mutant variants. As depicted in Figure 4A, SZ_A (D12A, G197D, Y369E, T372N, E375D, and I378R) with the most conservative amino acids exhibited superior extension compared to SZ_B (D12A, G197D, Y369K, T372N, E375D and I378R), SZ_C (D12A, G197D, T368I, Y369E, T372N and E375S), SZ_D (D12A, G197D, T368I, Y369K, T372Q, and E375S), and D12A in a 300 mM KCl environment. This is consistent with our hypothesis that conserved amino acid substitutions may be an effective strategy for enhancing salt tolerance. In comparison with Lys at the Y369 position in SZ_B, which showed the highest frequency on the HotSpot website, the conserved amino acid Y369E in SZ_A excelled in both extension activity and salt tolerance categories. Although SZ_C exhibited better binding affinity than SZ_A, the mutations E375D and I378R in SZ_A polymerase aided in the production of longer DNA products than E375S and T368I in SZ_C in the presence of 300 mM KCl (Figures 3E, 4A). Compared with SZ_C, Y369K in SZ_D included an extra positive charge on the lower edge of the substrate entrance, which skewed the position of the template, resulting in weaker template binding, as shown in Figure 6. We postulate that this may explain why SZ_D did not exhibit better incorporation attributes.

We demonstrated an innovative approach by seamlessly integrating a droplet-based microfluidic technique with conventional CSR for mutant site screening of polymerases with enhanced salt tolerance. By employing a combined evaluation of bioinformatics and biochemical assays, substitution of a regular site with a conserved amino acid was found to be an effective strategy for enhancing the salt tolerance of polymerases. Among all variants, we identified SZ_A as the most conserved amino acid, which exhibited favorable characteristics for nanopore sequencing, including enhanced salt tolerance, better processivity, and deficient exonuclease activity. Lastly, this methodology not only unlocks the regulatory sites of polymerase activity for salt tolerance enhancement but also introduces a novel strategy for sequence design through amino acid substitution. This advancement opens new possibilities in the field of biotechnology, unveiling innovative avenues for further exploration and application.

## Data availability statement

The data presented in this study are deposited in the NCBI GenBank repository under accession numbers OR420926-OR420931.

## Author contributions

YS: Conceptualization, Formal analysis, Funding acquisition, Investigation, Methodology, Visualization, Writing – original draft, Writing – review & editing. DK: Formal analysis, Methodology, Validation, Visualization, Writing – review & editing. JG: Validation, Writing – original draft. KF: Validation, Writing – original draft. YG: Formal analysis, Writing – original draft. QZ: Resources, Writing – original draft. SB: Resources, Writing – original draft. TH: Formal analysis, Writing – original draft. II: Resources, Supervision, Writing – review & editing. YH: Resources, Supervision, Writing – review & editing. HT: Resources, Supervision, Writing – review & editing.
