# Supplementary material for: Unraveling the salt tolerance of Phi29 DNA polymerase using compartmentalized self-replication and microfluidics platform
Source: Front Microbiol. 2023 Nov 7;14:1267196. doi: 10.3389/fmicb.2023.1267196 (PMC10661337; doi:10.3389/fmicb.2023.1267196)
Supplement: Supplementary file 1 [file Data_Sheet_1.docx]

Supplementary Material

Unraveling the salt tolerance of Phi29 DNA polymerase using compartmentalized self-replication and microfluidics platform

Yaping Sun^1^, Danny Hsu Ko^2^, Jie Gao^2^, Yaping Gao^1^, Kang Fu^2^, Qiwen Zhang^2^, Salem Baldi^2^, Tao Hong^2^, Igor Ivanov^2^, Yun He^1^* and Hui Tian^1^*

*** Correspondence:**

Corresponding Author: [hej@tsinghua-sz.org](mailto:hej@tsinghua-sz.org); [tianhui@tsinghua-sz.org](mailto:tianhui@tsinghua-sz.org)

# Establishment of mutagenesis libraries

1. The Phi29 polymerase gene was randomly mutated using the Beyotime Random Mutagenesis Kit (D0219M, Beyotime, China) or Mn^2+^. The reaction system and conditions are presented in Tables 1-3. Additionally, the forward and reverse primers used in the mutagenesis reaction library can be found in Supplementary Table 1.

Table 1. MnCl_2_ mutagenesis system

| Reagent | Volume (µl) | Final concentration |
| --- | --- | --- |
| MnCl_2_ (50 mM) | 0.2 | 0.2 mM |
| MgCl_2_ (50 mM) | 5 | 5 mM |
| dNTPs (10 mM) | 2 | 0.4 mM |
| Template | 20 ng |  |
| Primers mix (5 µM) | 4 | 0.4 µM |
| Unbalance (dATP+dCTP or dTTP+dGTP) (10 mM) | 1 | 0.2 mM |
| Taq polymerase buffer (10x) (B9014S, New England Biolabs) | 5 | 1x |
| Taq polymerase (M0273X, New England Biolabs) | 1 |  |
| ddH_2_O | Volumetric to a total volume of 50 μl | |

Table 2. Beyotime Random Mutation Kit

| Reagent | Volume (µl) | Final concentration |
| --- | --- | --- |
| Random mutation buffer (10x) | 5 | 1x |
| Mutation enhancer (10x) | 5 | 1x |
| dNTPs (10 mM） | 1.25 | 0.25 mM |
| Template | 350 ng |  |
| Primers mix (5 µM) | 2 | 0.2 mM |
| Random DNA polymerase | 1 |  |
| ddH_2_O | Volumetric to a total volume of 50 μl | |

Table 3. PCR reaction conditions

| Temperature | Time |  |
| --- | --- | --- |
| 95℃ | 2 min |  |
| 95℃ | 30 s | 14 cycles |
| 58℃ | 30 s |  |
| 68℃ | 2.5 min |  |
| 68℃ | 5 min |  |
| 4℃ | preservation | |

B. The phi29 gene fragment was recovered after random mutagenesis using Monarch PCR & DNA Cleanup kit (T1030L, New England Biolabs).

C. The recovered gene fragments (2.7 µg in total) were ligated to the pET30A vector (fragment:vector molar ratio = 3:1).

D. The plasmid was transformed into trans10 (CC96107-01, TOLOBIO, Wuxi, China) and transferred to LB medium containing kana antibiotics (final concentration 50 µg/mL) and incubated overnight at 37°C in a refrigerated incubator shaker (ZQLY-180ES, Shanghai Zhichu Instrument, China).

E. Plasmids are extracted the following day using a FastPure plasmid mini kit (DC201-01, Vazyme, Jiangsu, China). The plasmid obtained is the initial mutant library.

# Preparing of cells

A. 1 µg of library plasmids were transformed into BL21 receptor cells a day in advance and incubated overnight at 37°C in a shaker comprised of 5 mL of LB medium containing Kana antibiotics.

B. In the next morning, 100 µL of bacterial broth was transferred 1:100 into 10ml of LB medium containing Kana antibiotics and incubated for 2 hrs at 37°C in a shaker for the broth to reach an exponential growth phase (OD600 = 0.4 - 0.6).

C. The culture was induced using IPTG and continued for 4 hrs at 37°C.

D. At the end of the incubation, 5 mL of the induction product was centrifuged at 2500 rcf for 10 min at 4°C. The supernatant was discarded and used in 1X Tango buffer (1X Tango buffer (BY5, Thermo Scientific, USA) formulation: 30 mM Tris-acetate, (pH 7.9 at 37°C), 10 mM Mg- acetate, 66 mM K-acetate, 0.1 mg/mL BSA) washed twice.

E. The washed cells were resuspended in a 1X Tango buffer, transferred to a 1.5 mL EP tube, 53 μl (final concentration 0.5 mg/mL) of Lysozyme (10 mg/mL, A610308-0005, Sangon Biotech, Shanghai, China) was added and left at room temperature for 5-10 min.

# Preparing of carrier oil

The carrier oil was prepared in a beaker (DS-SNSB-01, Shubo) according to Table 4 and mixed continuously for 10 min at 4°C on a magnetic stirrer at 1500 rpm, and left to defoam at the end of mixing. Afterwards, 700 µL of the carrier oil was transferred to a 2 mL EP tube.

Table 4: Oil phase formulations

| Reagent | Volume (µl) | Final concentration |
| --- | --- | --- |
| ABIL EM 90 (Degussa) | 200 μL | 2 % |
| Triton X-100 (A110694-0500, Sangon Biotech, Shanghai, China) | 5.5 μL | 0.055 % |
| Mineral oil (M5904-500ML, Sigma-Aldrich, St.Louis, MO, USA) | 9800 μL |  |

# Preparing the CSR mix

The CSR mix was prepared according to Table 5.

| Reagent | Volume (µl) | Final concentration |
| --- | --- | --- |
| 10X Tango buffer | 30 μL | 1X Tango buffer |
| 1 mM Exo-resistant random primer mix | 7.5 μL | 25 μM |
| 10 μM of Specific primer-forward/reverse mix (Supplementary Table 1) | 9 μL | 0.3 μM |
| 10 mM dNTP analogues | 30 μL | 1 mM |
| E coli | 50 μL |  |
| H_2_O | 173.5 or 143.5 μL |  |
| KCl (3M） | 0 or 30 μL | 300 mM |
| Total | 300 μL |  |

# Building the emulsion mass

1. Place 700 uL of the carrier oil based on the reagent mix described above into a magnetic stirrer and stir continuously for 2 min at 1500 rpm at 4°C.
2. Add dropwise 0.3 mL of CSR mixture to the carrier oil with continuous stirring for 2 min. Mix well and place in a -80°C refrigerator.

# CSR product purification

1. Freeze the emulsion mixture at -80°C for 30 min, then thaw in a 30°C water bath for 5 min. Repeat 5 times to break the cells.
2. Place the emulsion containing both the frozen and thawed mixture in a 30°C water bath (HH-2, Yiheng biotech, Shanghai, China) for 2 hrs.
3. Transfer the emulsion to a 1.5 mL centrifuge tube and centrifuge for 5 min at 13, 000 g. Remove the supernatant.
4. Add an equal volume of phenol:chloroform:isoamyl alcohol (25:24:1) to the DNA solution for purification and mix thoroughly on a vortex mixer.
5. Centrifuge the mixture at 12,000 r/min for 10 min and remove the upper aqueous phase containing DNA to a new centrifuge tube. If there are white precipitates present between the junction of the aqueous and organic phase, re-extracted the aqueous solution until there are no visible precipitates at the junction between the two phases.
6. Transfer the upper aqueous phase containing DNA to a new centrifuge tube. Add 1/10th the volume of sodium acetate (BL541A, Biosharp, Hefei, China) and mix thoroughly. Add pre-cooled anhydrous ethanol to the tube (> 2.5 V), mix by inversion and store at -80 °C for 30 min or overnight.
7. Centrifuge at 12000 rpm/min for 10 min and discard the supernatant.
8. Add 1 mL of 75% ethanol to the precipitate and centrifuge at 12,000 rpm for 5 min, discard the supernatant.
9. Add 20 μL of water to the centrifuge tube containing the dried DNA.
10. Run alkaline agarose gel to verify the results after purification.
