# Supplementary figures and images for: Unraveling the salt tolerance of Phi29 DNA polymerase using compartmentalized self-replication and microfluidics platform
Source: Front Microbiol. 2023 Nov 7;14:1267196. doi: 10.3389/fmicb.2023.1267196 (PMC10661337; doi:10.3389/fmicb.2023.1267196)

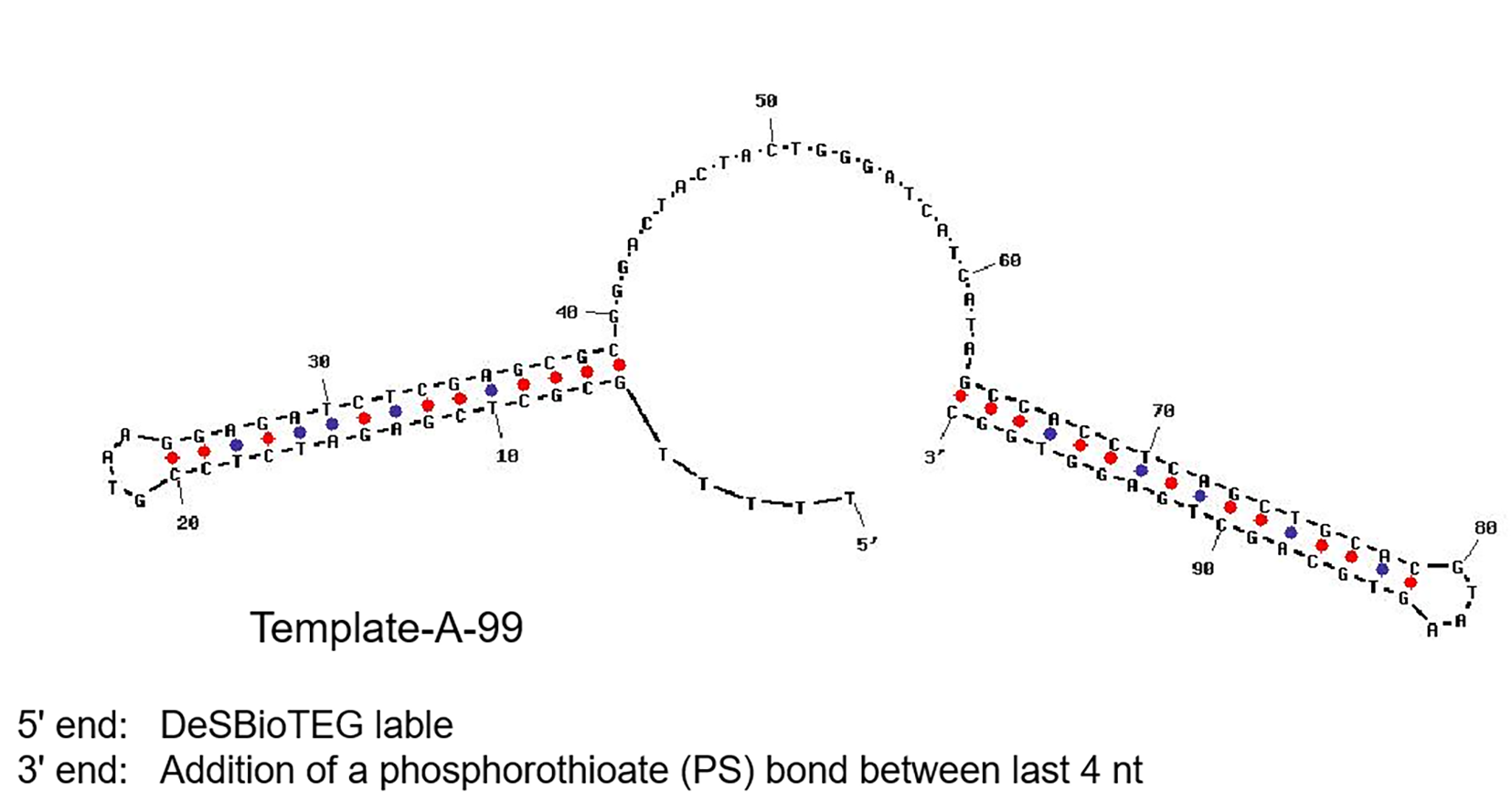

Supplement: Supplementary file 2 [file Image_1.TIF]

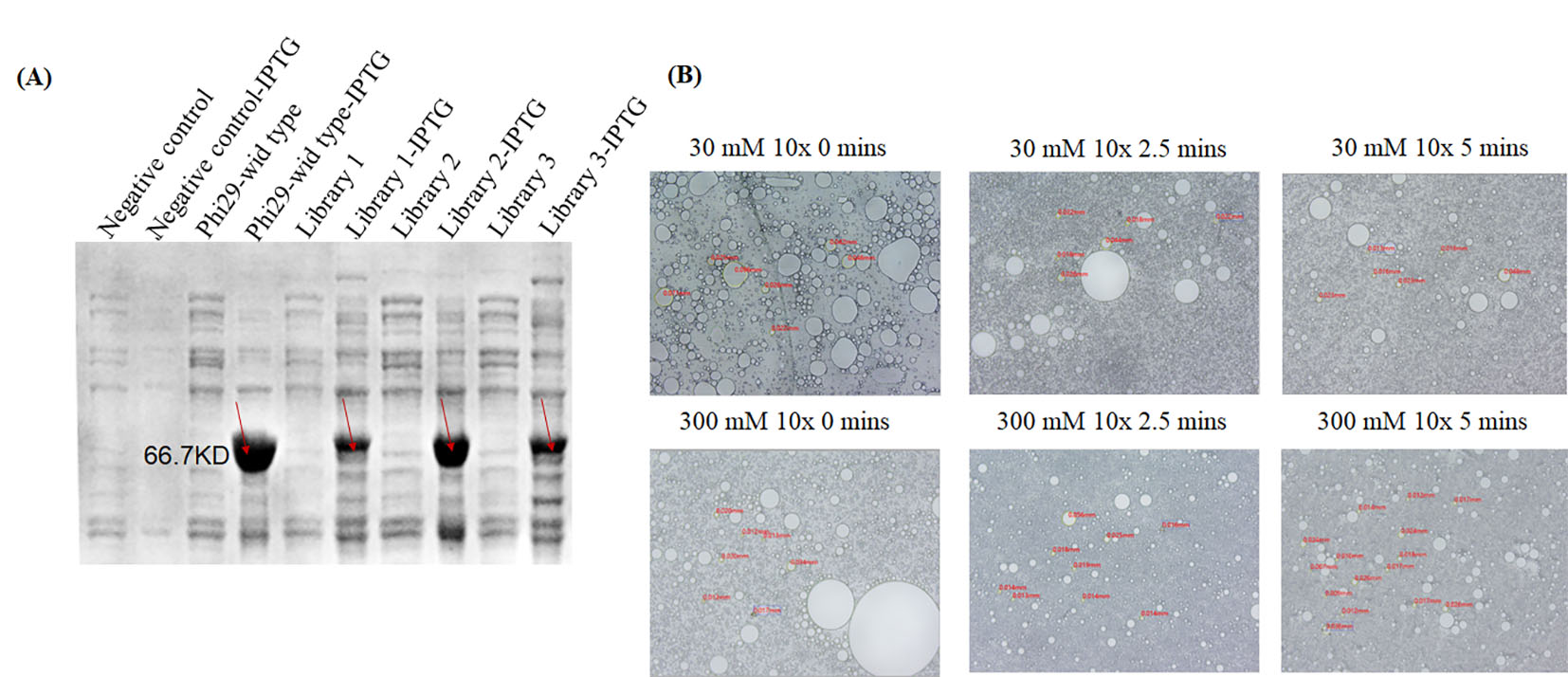

Supplement: Supplementary file 3 [file Image_2.JPEG]

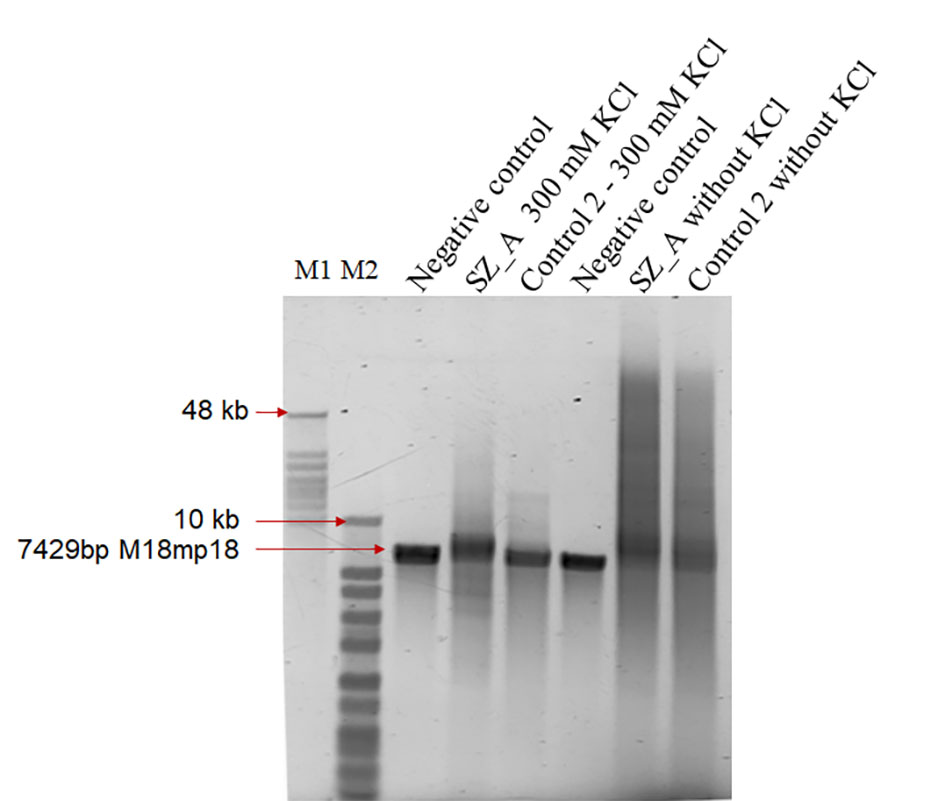

Supplement: Supplementary file 4 [file Image_3.JPEG]
